# Use of a trigger tool to detect adverse drug reactions in an emergency department

**DOI:** 10.1186/s40360-017-0177-y

**Published:** 2017-11-15

**Authors:** Silvana Maria de Almeida, Aruana Romualdo, Andressa de Abreu Ferraresi, Giovana Roberta Zelezoglo, Alexandre R. Marra, Michael B. Edmond

**Affiliations:** 1 Pharmacist, Hospital Israelita Albert Einstein, Avenida Albert Einstein, 627 – bloco E, 2° andar, Morumbi, São Paulo, 05651-901 Brazil; 2 Division of Medical Practice, Hospital Israelita Albert Einstein, São Paulo, Brazil; 3 Office of Clinical Quality, Safety and Performance Improvement, University of Iowa Hospitals and Clinics, Iowa City, IA USA

**Keywords:** Trigger tool, Pharmacovigilance, Adverse drug reactions, Medication, Hospital, Methodology, Pharmacy, Quality assurance, Reports, Emergency room

## Abstract

**Background:**

Although there are systems for reporting adverse drug reactions (ADR), these safety events remain under reported. The low-cost*,* low-tech trigger tool method is based on the detection of events through clues, and it seems to increase the detection of adverse events compared to traditional methodologies. This study seeks to estimate the prevalence of adverse reactions to drugs in patients seeking care in the emergency department.

**Methods:**

Retrospective study from January to December, 2014, applying the Institute for Healthcare Improvement (IHI) trigger tool methodology for patients treated at the emergency room of a tertiary care hospital.

**Results:**

The estimated prevalence of adverse reactions in patients presenting to the emergency department was 2.3% [CI_95_ 1.3% to 3.3%]; 28.6% of cases required hospitalization at an average cost of US$ 5698.44. The most common triggers were hydrocortisone (57% of the cases), diphenhydramine (14%) and fexofenadine (14%). Anti-infectives (19%), cardiovascular agents (14%), and musculoskeletal drugs (14%) were the most common causes of adverse reactions. According to the Naranjo Scale, 71% were classified as possible and 29% as probable. There was no association between adverse reactions and age and sex in the present study.

**Conclusions:**

The use of the trigger tool to identify adverse reactions in the emergency department was possible to identify a prevalence of 2.3%. It showed to be a viable method that can provide a better understanding of adverse drug reactions in this patient population.

## Background

According to the World Health Organization (WHO), an adverse reaction to drugs is the harmful and unintentional reaction to the use of medications, which occurs at doses normally used in humans for the prophylaxis, diagnosis, or treatment of diseases or to modify a physiological function [1, 2]. Adverse drug reaction (ADR) is the fifth leading cause of death among Americans, surpassed only by heart disease, stroke, cancer, and lung diseases [3, 4]. The economic consequences are still not well elucidated, but there are two points to consider: the cost related to treatment and the cost related to prevention. The cost estimate for treatment in the United States reaches $130 billion per year [3–5]. Estimates in France suggest that up to 123,000 patients per year require medical treatment due to ADRs and these patients often require hospitalization [6–12].

Although there are systems to report ADRs, there is consistent under reporting. Therefore, the true rates of ADRs are difficult to determine, and cases leading to hospitalization or death may not be captured. Additionally, ADRs are important to detect and to report because the majority of them are considered preventable [13–15].

The Institute for Healthcare Improvement (IHI) trigger tool is a low-cost, low-tech method for detecting adverse events and adverse reactions through clues (triggers) such as: the use of antidotes, antiemetics, or antidiarrhea agents, variations in relevant laboratory tests such as the prothrombin time, INR (international normalized ratio), plasma levels of low therapeutic index drugs (phenytoin, carbamazepine, etc.), signs and symptoms such as cutaneous rash, and patient transfer to intensive care units. This technique seems to increase the detection rate of adverse events by a factor of approximately 50 compared to traditional methodologies [4, 14–17].

The *Hospital Israelita Albert Einstein*, accredited by the JCI (Joint Commission International) is heavily involved in patient safety issues and has a pharmacovigilance program that monitors adverse events and ADRs; however, it suffers from a lack of accurate information about the actual number of adverse reactions and from a lack of specific monitoring indicators.

The objective of the present study is to estimate the prevalence of suspected adverse reaction to drugs in patients seeking treatment in the emergency room – (ER), describing the causes and the related factors, using the trigger tool method.

## Methods

**Fig. 1:**
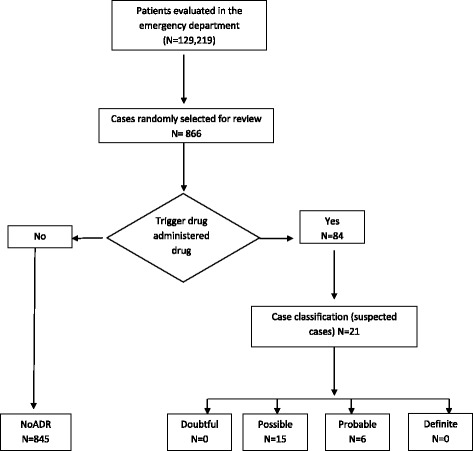
Study flow chart

A retrospective study was conducted in the *Hospital Israelita Albert Einstein*, a 600-bed hospital located in the city of São Paulo, Brazil. Its emergency department treats 400 patients daily. The study gathered data from 866 randomly selected patients treated in the emergency department (Fig. 1) during the time period January 1, 2014 to December 31, 2014. This study was approved by Institutional Research Ethics Committee.

Inclusion criteria included the administration of specified trigger medications, drugs that might be given in response to suspected adverse reactions to other drugs. Each trigger medication administration was identified via review of the electronic medical record. The following medications were used as triggers: antihistamines and anti-allergy medications that can be used in cases of anaphylactoid or allergic reactions: diphenhydramine, fexofenadine, methylprednisolone, hydrocortisone, and hydroxyzine. Medications used to reverse the action of other drugs were also used as triggers: phytomenadione, used to reverse the action of oral anticoagulants such as warfarin; acetylcysteine, which, among other things, is used in cases of paracetamol and acetaminophen overdose; naloxone, a medication used to reverse the action of opioids (morphine, methadone, fentanyl, codeine); and flumazenil, a medication used to reverse the action of benzodiazepines (alprazolam, bromazepam, clobazam, clonazepam, diazepam, flunitrazepam, midazolam, and lorazepam). Emergency department patients that did not receive a trigger medication were excluded. Among the triggers used to detect adverse events, we chose drugs that are normally used to reverse adverse reactions in the hospital. We did not use other trigger medications related to diagnostic exams, use of specific treatments like dialysis or blood transfusion, or surgical procedures, transfer to critical care units, or activation of a rapid response team.

The Naranjo Scale was used to assess causality, and the cases were classified as: doubtful, possible, probable, or definite [18]. The patients were stratified into three age groups: under the age of 18 years, between 18 and 60 years, and those over 60 years. The International Code of Diseases – ICD 10 [19] was used to classify the diagnoses for the emergency department visit. The cost was calculated for the visits of patients involved in the suspected events. It included all procedures, medications and daily hospitalization costs. Inter-rater reliability regarding the identification of suspected ADRs was determined using the Kappa coefficient [20, 21]. The study was exempted from the requirement for informed consent after an evaluation by the Committee for Ethics in Research.

### Statistical analysis

Categorical variables were described by absolute frequencies and percentages, and the numerical variables, by median and interquartile range (IQR), in addition to the minimum and maximum values. The sample size was calculated based on the proportion of suspected adverse reaction to drugs in patients seeking treatment in the emergency room. Assuming that the rate of occurrence of ADR is 2.5%, a sample size of 865 patients produces a two-sided 95% confidence interval with a width equal to 2.0%.

To estimate the prevalence of visits to the emergency room due to suspected adverse drug reactions, a model of generalized estimating equations (GEE) was set, considering the correlation between measurements on the same patient on different visits. The results of the model were presented by adjusted proportion and a 95% confidence interval. The analyses were performed using SPSS version 24 with the significance level set at 5% [22–25].

## Results

### Population characteristics

The age of patients from the emergency department randomly selected for review (*N* = 866) at the time of the visit ranged between 0 (newborn) and 98 years, and half of them were under 32 (Q1 = 10 years and Q3 = 45 years). Stratifying by age, 34.6% were under 18 years, 55.3% were between 18 and 59 years, and 10.0% were 60 years or over, with a 51.0% females and 49.0%, males.

There were 44 evaluations conducted by two raters to assess inter rater reliability. As for the presence of adverse reactions, the raters agreed in 95.4% of the cases, with 0.906 coefficient of agreement (standard deviation 0.065, *p* < 0.001). As for the Naranjo Scale, the raters agreed in 93.2% of the cases, with a coefficient of agreement of 0.877 (standard deviation 0.068, *p* < 0.001).

Of the total population analyzed (*n* = 866), 9.7% of patients (*n* = 84) were prescribed medication considered to be a trigger, with 16 (19%) requiring hydrocortisone, 16 (19%) methylprednisolone, 14 (16.6%) requiring fexofenadine, 8 (9.5%) requiring hydrocortisone plus diphenhydramine, 5 (5.9%) hydroxyzine, 4 (4.8%) hydrocortisone plus fexofenadine, 3 (3.6%) requiring diphenhydramine, 3 (3.6%) diphenhydramine plus methylprednisolone, 3 (3.6%) hydrocortisone plus diphenhydramine plus fexofenadine, 2 (2.4%) fexofenadine plus methylprednisolone, 2 (2.4%) hydrocortisone plus methylprednisolone, 1 (1.2%) requiring acetylcysteine, 1 (1.2%) requiring activated charcoal, 1 (1.2%) requiring phytomenadione, 1 (1.2%) requiring diphenhydramine plus fexofenadine, 1 (1.2%) requiring hydrocortisone plus fexofenadine plus methylprednisolone, 1 (1.2%) requiring hydrocortisone plus diphenhydramine plus hydroxyzine, 1 (1.2%) requiring hydrocortisone plus diphenhydramine plus methylprednisolone and 1 (1.2%) flumazenil (Table 1).

**Table 1:** Distribution of trigger drugs used on the population studied and in cases of ADR

Trigger drugs	Total population	Suspected cases
	n(%)	n(%)
Diphenhydramine	3 (3.6)	1 (4.8)
Diphenhydramine / fexofenadine	1 (1.2)	1 (4.8)
Difenidrin / Methylprednisolone	3 (3.6)	1 (4.8)
Fexofenadine	14 (16.6)	2 (9.5)
Fexofenadina / Methylprednisolone	2 (2.4)	1 (4.8)
Phytomenadione	1 (1.2)	1 (4.8)
Hydrocortisone	16 (19)	1 (4.8)
Hydrocortisone / Diphenhydramine	8 (9.5)	5 (23.8)
Hydrocortisone / Diphenhydramine/ fexofenadine	3 (3.6)	1 (4.8)
Hydrocortisone / Diphenhydramine / Methylprednisolone	1 (1.2)	1 (4.8)
Hydrocortisone / fexofenadine	4 (4.8)	2 (9.5)
Hydrocortisone / fexofenadine / Methylprednisolone	1 (1.2)	1 (4.8)
Hydroxyzine	5 (5.9)	3 (14.3)
Activated charcoal	1 (1.2)	
Acetylcysteine	1 (1.2)	
Flumazenil	1 (1.2)	
Hydrocortisone / Diphenhydramine / Hydroxyzine	1 (1.2)	
Hydrocortisone / Methylprednisolone	2 (2.4)	
Methylprednisolone	16 (19)	
Total	84(100)	21(100)

The most frequent diagnoses for which the offending drug was prescribed (the underlying diseases identified by the physician who attended the patients) were related to otorhinolaryngology (19.3%), gastroenterology (11.8%), lung disease (11.8%), and neurology (7.9%). The majority of patient did not require hospitalization (81.8%).

### Suspected adverse reaction

The rate of occurrence of ADR was 2.4% (*n* = 21) in the sample of 866 visits to the ER (Fig. 1); however, one patient had two visits and both due to the occurrence of an ADR event. Thus, it was necessary to adjust the ratio to monitor this duplication.

Considering the repetition of events in one patient, the estimated rate is 2.3% (95% CI from 1.3% to 3.3%).

The ADRs detected were: urticaria (*n* = 4), pruritus (*n* = 2), respiratory problem (*n* = 2), angioneurotic edema (*n* = 2), anxiety disorder (*n* = 1), prurigo/cutaneous eruption (*n* = 1), fatigue (*n* = 1), hordeolum (*n* = 1), bleeding/hematoma (*n* = 1), skin eruption (*n* = 1), ocular edema (*n* = 1), tongue edema (*n* = 1), dermatitis (*n* = 1), allergy not specified (*n* = 1), restlessness (*n* = 1).

By adjusting the model to estimate the prevalence of visits to the emergency department due to suspected adverse reactions, we found the estimated prevalence to be 2.31% [1.31% to 3.31%]. There was no association with age (*p* = 0.248) or gender (*p* = 0.901). The prevalence was 2.3% for males and 2.4% for females. With regards to age, the prevalence of ADR was 1.3% (CI_95%_0.5% - 3.1%) in patients younger than 18 years, 2.7% (CI_95%_1.5% - 4.5%) in patients between 18 and 59 years, and 3.4% (CI_95%_1.0% - 8.9%) in patients aged 60 years or older.

Among the most frequent diagnoses at the time of presentation for the ADR in this group were upper airway infections (14.2%), unspecified dermatitis (9.5%), urticaria (9.5%), unspecified allergies (9.5%), and unspecified acute bronchitis (9.5%) (Table 2).

**Table 2:** Characteristics of patients treated in the emergency room forADR

Presented with suspected adverse reaction	n(%)
No	845 (97.6)
Yes	21 (2.4)
Total	866 (100)
Assessment –Naranjo Scale	n(%)
Definite	0
Probable	6 (28.6)
Possible	15 (71.4)
Doubtful	0
Total	21 (100)
Total	21 (100)
Classification of medications causing ADRs	n(%)
Anti-infectives	4 (19.0)
Cardiovascular	3 (14.3)
Musculoskeletal	3 (14.3)
Not recorded	2 (9.5)
Digestive tract and metabolism	1 (4.8)
Personal hygiene and cleaning/cosmetic products	1 (4.8)
Hematologic	1 (4.8)
Respiratory	1 (4.8)
Neurologic + Endocrine	1 (4.8)
Neurologic + Cardiovascular + GI	1 (4.8)
Neurologic + Cardiovascular	1 (4.8)
Musculoskeletal + Personal hygiene and cleaning/cosmetic products	1 (4.8)
Musculoskeletal + Anti-infective	1 (4.8)
Total	21 (100)
Primary diagnosis of patients with ADR assigned by the provider	n(%)
Acute upper respiratory infections	3 (14.2)
Acute bronchitis	2 (9.5)
Dermatitis	2 (9.5)
Urticaria	2 (9.5)
Other and unspecified allergy	2 (9.5)
Anxiety disorder	1 (4.8)
Hordeolum of eyelid	1 (4.8)
Pneumonia	1 (4.8)
Dyspepsia	1 (4.8)
Gastrointestinal hemorrhage	1 (4.8)
Other prurigo	1 (4.8)
Mucocutaneous lymph node syndrome [Kawasaki]	1 (4.8)
Back Pain	1 (4.8)
Urinary tract infection	1 (4.8)
Angioneurotic edema	1 (4.8)
Total	21 (100)
Outcome (*n* = 21)	n(%)
Discharge	15 (71.4)
Admission	6 (28.6)
Length of stay (days)	n(%)
0	16 (76.2)
1	1 (4.8)
2	1 (4.8)
3	1 (4.8)
11	1 (4.8)
12	1 (4.8)
Total	21 (100)

Among patients who received trigger drugs, 25% (*n* = 21) had a suspected ADR; 23.8% (*n* = 5) were identified through the use of hydrocortisone plus diphenhydramine; 14.3% (*n* = 3) were identified through the use of hydroxyzine; 9.5% (n = 2) with fexofenadine and 9.5% (*n* = 2) hydrocortisone plus fexofenadine (Table 1).

As for the suspected medications causing adverse reactions in the 21 patients, 19% (*n* = 4) were anti-infectives, 14.3% (*n* = 3) were cardiovascular drugs, and 14.3% (*n* = 3) were drugs for the musculoskeletal system. The positive predictive value of the administration of trigger drugs was 25% (21/84%).

### Outcome

Applying the Naranjo Scale to evaluate the probability of an adverse drug reaction, we found that 15 (71%) were possible and 6 (29%) with probable ADRs. There were no reaction cases classified as doubtful or definite, and there were no fatal cases (Table 2).

Of the 21 ADRs observed in the sample, 28.6% required hospitalizations that lasted from 1 to 12 days (Table 2).

As for the costs associated with outpatient and inpatient visits, a total cost of US$38,020.60 was seen for the 21 patients—an average of US$1810.50 per patient with suspected ADR, whereas patients who were hospitalized incurred a total cost of US$34,193.10, an average of US$5698.84 per patient.

## Discussion

Although there are various reporting systems for adverse reactions and adverse events, there is consistent under-reporting of ADR, and it has been shown that many ADRs represent known interactions and are likely to be preventable [13–15]. In this study, drugs considered as triggers were used to identify suspected adverse reactions of patients presenting to the emergency room, and a 2.3% prevalence of adverse drug reactions was identified in the population studied. Several studies conducted to identify adverse events among patients in the emergency room using various other methodologies found incidence rates between 0.9 and 3.9%, while another study found 4.7% [26–28]. In spite of studies indicating that the number of visits to emergency departments due to an ADR is higher in elderly patients [29], the present study did not find an association between cases of suspected ADRs and age. A recent 2013–2014 study of surveillance data in the United States [28] identified that the most common cause of these events were related to anti-infectives, similar to what we found in the present study. Anticoagulants, diabetes agents and opioid analgesics were implicated as other commons reasons for emergency department visits related to ADRs in the national survey [28], while the present study found cardiovascular drugs and musculoskeletal drugs as common medications causing ADRs, with these categories also appearing in other studies [26–31].

Another American study found their most frequent diagnoses associated with the adverse reaction to be: skin conditions, gastrointestinal illnesses, and neurological conditions [27]. In the present study, the most frequent diagnoses were: upper airway infections, unspecified acute bronchitis, unspecified dermatitis, urticaria, and unspecified allergies.

Applying the Naranjo Scale to evaluate the causal relationship of the occurrences of suspected ADRs, 15 (71%) were found with a possible causal relationship and 6 (29%) were found to be probable, which differed from the findings in a British study in which the majority of the cases were considered probable (approximately 69%) followed by possible (29%), and definite (between 0.7 and 2.9%) [13]. The present study had no reaction cases classified as definite or doubtful.

A population-based study in patients older than 16 years assessing the need for hospitalization identified that 80% of ADR cases led to hospitalization [13]. In another study in elderly patients, 21.6% were hospitalized due to adverse reactions [32]. In the United States, 27.3% of emergency department visits for ADR resulted in hospitalizations [28]. In the present study, we found that 28.6% of the ADR cases needed hospitalization and there were no fatal cases.

As for the costs associated with patients’ visits, one study gave an average of US$8888 per admission, and in another study of an elderly population, hospitalization due to severe adverse reactions represented an additional cost of US$11 million per year, while in another study the cost was US$2262 per ADR [28, 32, 33]. In the present study, there was a total cost of US$38,020.60 for the 21 patients, an average of US$1810.50 per patient with suspected ADR, whereas patients who were hospitalized had a total cost of US$34,193.10, an average of US$5698.84 per patient hospitalized.

One of the limitations of the present study was the use of trigger drugs alone to identify suspected ADRs [28]. No searches for abnormal results from laboratory tests were included. It needs to be stated that not all ADRs require medications that would be identified as trigger drugs, so in this study such ADRs would not have been detected. Another limitation is that, since it was a retrospective study, there was no access to other information from the patients, such as the use of herbal medicines and other alternative therapies before the event; the information was limited to what was described in the medical charts of these patients. Although the sample size could be assumed as a limitation of this study, the most important point of the study is that we were able to detect ADRs in the emergency department by the trigger tool, and we were able to prevent new ones and reduce harm.

## Conclusions

We conclude that the application of trigger drugs to identify adverse reactions in the emergency department is a viable method and can be used to better understand the adverse drug reactions of patients treated in the emergency room and to direct actions related to pharmacovigilance in this sector.

## Abbreviations

ADR: Adverse drug reaction
ER: Emergency room
ICD 10: The International Code of Diseases
IHI: Institute for Healthcare Improvement
INR: International normalized ratio
JCI: Joint Commission International
WHO: World Health Organization
